# 3D Online Submicron Scale Observation of Mixed Metal Powder's Microstructure Evolution in High Temperature and Microwave Compound Fields

**DOI:** 10.1155/2014/684081

**Published:** 2014-03-11

**Authors:** Dan Kang, Feng Xu, Xiao-fang Hu, Bo Dong, Yu Xiao, Ti-qiao Xiao

**Affiliations:** ^1^CAS Key Laboratory of Mechanical Behavior and Design of Materials, University of Science and Technology of China, Hefei 230026, China; ^2^Shanghai Institute of Applied Physics, Chinese Academy of Sciences, Shanghai 201204, China

## Abstract

In order to study the influence on the mechanical properties caused by microstructure evolution of metal powder in extreme environment, 3D real-time observation of the microstructure evolution of Al-Ti mixed powder in high temperature and microwave compound fields was realized by using synchrotron radiation computerized topography (SR-CT) technique; the spatial resolution was enhanced to 0.37 **μ**m/pixel through the designed equipment and the introduction of excellent reconstruction method for the first time. The process of microstructure evolution during sintering was clearly distinguished from 2D and 3D reconstructed images. Typical sintering parameters such as sintering neck size, porosity, and particle size of the sample were presented for quantitative analysis of the influence on the mechanical properties and the sintering kinetics during microwave sintering. The neck size-time curve was obtained and the neck growth exponent was 7.3, which indicated that surface diffusion was the main diffusion mechanism; the reason was the eddy current loss induced by the external microwave fields providing an additional driving force for mass diffusion on the particle surface. From the reconstructed images and the curve of porosity and average particle size versus temperature, it was believed that the presence of liquid phase aluminum accelerated the densification and particle growth.

## 1. Introduction

A lot of the metals and alloys with excellent properties are prepared by powder metallurgy at high temperature [1, 2]. Microwave technology [3, 4] can not only provide high-temperature environment rapidly and uniformly but also make the materials have excellent mechanical properties (e.g., higher strength, hardness, ductility, and toughness) because of the combined effects of high temperature and electromagnetic fields. Since the first full sintering of powdered-metal [5] in 1999, microwave sintering has caused widespread concern and has become a new approach to prepare the high-performance metals and alloys. The properties of metals and alloys are greatly influenced by microstructures; consequently, high spatial resolution observations of the metal powder's microstructure evolution in high temperature and microwave compound fields are indispensable. The methods of observation, such as TEM and SEM, have been used for high spatial resolution online experiments in extreme environments (e.g., high temperature, high radiation, high pressure, etc.), but only the information of surface or two-dimensional (2D) section can be obtained. For the morphology, distribution, and scale of microstructures in three dimensions (3D) that control the properties, in summary, 3D online high spatial resolution characterization is more real and effective.

Synchrotron radiation X-ray computed tomography (SR-CT) technique is such a powerful imaging tool developed in recent years [6, 7]. It has been used in physical, chemical, and biological researches for its ability to image structure in 3D with high spatial resolution at macroscopic to submicroscopic scales; it is nondestructive and can realize the observation of microstructure evolution under extreme conditions in a real-time way [8, 9]. Currently, few scholars have carried out the in situ investigation of microwave sintering by the SR-CT technique. In our research group, in situ studies on the microwave sintering of metals have been carried out for the first time. However, due to the limitation of experimental apparatus and methods, it is difficult to increase the resolution yet it is essential.

In this paper, 3D microstructure characterization of Ti-Al metal powder mixture in high temperature and microwave compound fields was studied in situ, and the spatial resolution was firstly increased to 0.37 *μ*m/pixel. Phase recovery method was used to get high-contrast qualitative microtomography, and microstructure evolution during sintering was clearly distinguished from 2D and 3D reconstructed images, such as sintering neck size, porosity, and particle size of the sample. The size of the sintering neck in each cross section image was calculated by using watershed algorithm, and typical sintering parameters during sintering were presented for quantitative analysis of the influence on the mechanical properties and the sintering kinetics during microwave sintering.

## 2. Experiment

### 2.1. Brief Introduction of SR-CT Technique and Microwave Equipment

The SR-CT technique is a testing method by which the specimen passed through by synchrotron radiation. X-ray is placed on a rotation device and the projection images of the specimen are received by an X-ray charge-coupled device (CCD). One projection image is collected each time when the specimen turns for an angle. After obtaining a set of projection data, reconstruction algorithm is used to obtain the internal microstructure of the sectional images. The 3D images of the microstructure can be obtained from a series of sectional images.

### 2.2. Experimental Procedure

In the experiment, Al and Ti were selected because they were more commonly used in high-performance metals and alloys. The elemental Al and Ti powders were 300~600 mesh and mixed in the ball mill with alcohol for 10 hours to prepare 87Al-13Ti (wt.%) and then dried in a vacuum oven. The mixed alloy powder was filled into a quartz tube with an inner diameter of 260 *μ*m. The microwave sintering experiment was carried out on the BL13W1 beamline at Shanghai Synchrotron Radiation Facility (SSRF, China); the energy of the beam ranged from 8 to 72 KeV. Considering the X-ray absorption coefficient of Al and Ti, an X-ray with 26 KeV was applied.

**Figure 1:**
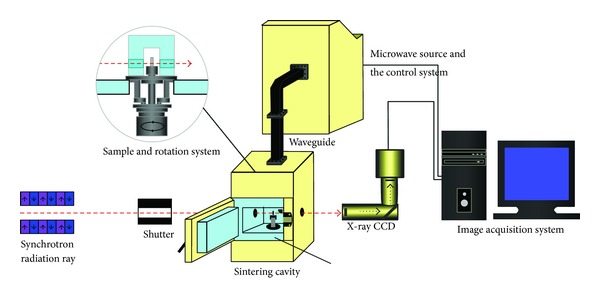
The schematic diagram of the SR-CT experiment system of microwave sintering.

A set of unique equipment specifically for the 3D imaging of samples using synchrotron of X-ray had been designed (Figure 1); the power of the microwave facility could be regulated continuously from 0 to 3 KW; online observation at very high temperature (above 1500°C) could be achieved. The system was capable of maintaining 3D tomograms at high spatial resolution to image structural details at high spatial resolution scale.

Due to the sampling capacity limit of CCD target surface, size of the view field would decrease with the improvement of the resolution; therefore, precise positioning of the sample was critical to achieve high resolution SR-CT experiment. The relationship between field of view size and resolution is shown in Table 1.

**Figure 2:**
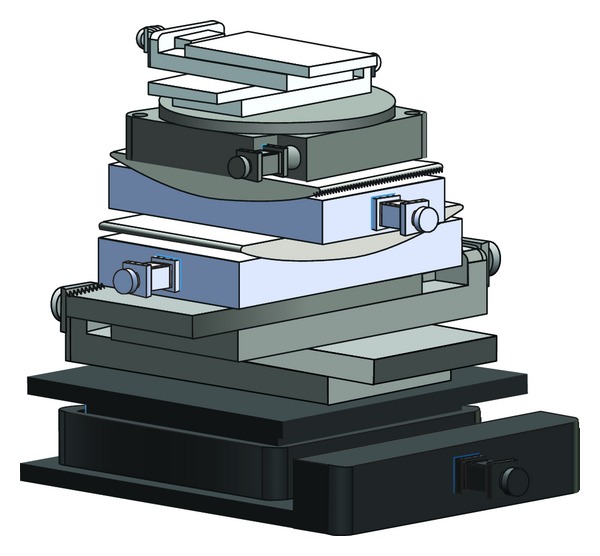
Structure of multidimensional high-precision translation and rotation equipment.

In order to carry out high resolution experiments, we developed a special multidimensional high-precision translation and rotation equipment; the design is shown in Figure 2. This device can adjust the sample to ensure it is rotating in the field of view, and it is independent from the cavity; the disturbance caused by the vibration of the cavity is eliminated. In order to guarantee the quality of projections, a loop control system of image acquisition and storage was designed to prevent exposure and image acquisition when the turntable was rotating. Based on this system, we have achieved the SR-CT experiment with resolution of 0.37 *μ*m/pixel successfully.

## 3. Results and Discussion

**Figure 3:**
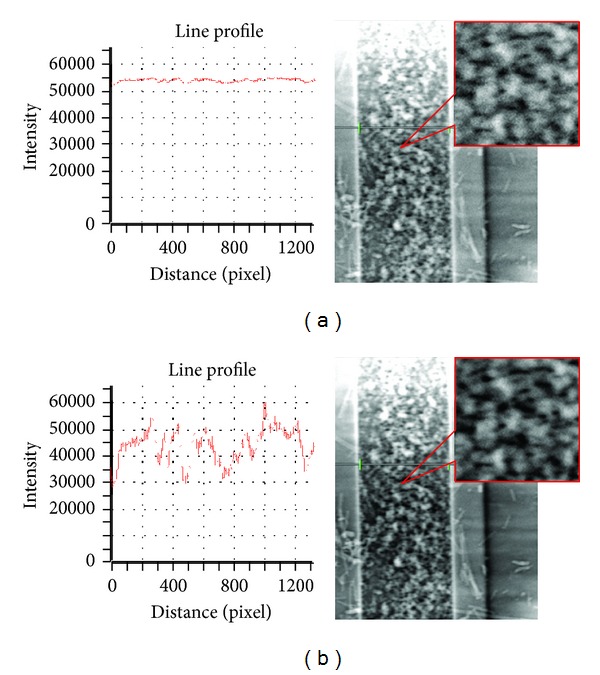
The intensity of projection image. (a) Original projection image. (b) Projection image after phase.

When there is a certain distance between CCD and the sample, phenomenon of absorption and phase shift occur simultaneously, the information received by CCD includes both absorption and phase information, and the phase contrast image cannot directly reflect the phase and structural information of the sample. X-ray propagation-based phase contrast CT (PPCT) method [10, 11] was used to get high-contrast qualitative microtomography (especially the microtomography of weakly absorbing samples), that is, edge enhancement. The microstructure boundaries become clear, as shown in Figure 3. This method is extremely useful for high-resolution experiments.

**Figure 4:**
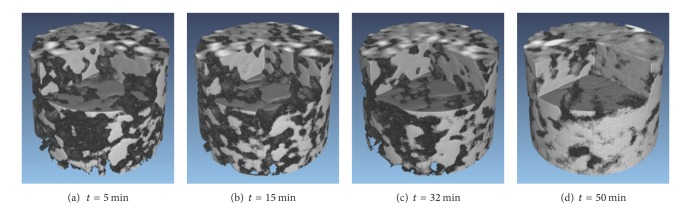
3D images of the microstructure at different sintering times.

The exposure time would be longer and more projectors would be required in high resolution SR-CT experiment, which led to significant changes in microstructure in the same group of projections, and because the intensity of X-ray decreased with time, the background intensity of projections would be inconsistent. Therefore, in order to increase data acquisition speed, the number of projections would be reduced. Using the inverse projection method could acquire high-quality reconstructed images with fewer projections. By using this method, a series of cross-sectional images at different sintering times was produced, from which 3D images were obtained by applying a 3D rendering algorithm. The 3D reconstructed images of the same microstructure at different sintering times are shown in Figure 4. Various conventional sintering phenomena can be observed clearly from the figure, such as the sintering necks form and grow; the interconnected pores become isolated and smaller, with the annexation of small particles; the particle size becomes larger; the sample appears to be dense. It is apparent that the combined effect of electromagnetic and high temperature field led the sintering phenomena to be more rapid and intense, especially the particle surfaces smoothing quickly, which may be caused by the “nonthermal effect” of microwave fields such as the increase of eddy current [12] on the particles' surface and the interfacial polarization between the grain surface and the pore.

Sintering necks which initiate and grow during sintering connect loose powders to form a solid metal material. The tensile strength of the metals can be significantly improved by improving the sintering neck structure. Neck growth is a basic phenomenon of sintering, and it plays an important role in determining the main diffusion mechanism and calculating the diffusion coefficient of the material [13]. Therefore, in order to research the kinetics mechanism of sintering neck growth, the watershed algorithm was used to extract the neck size; areas with different gray represent different particles; the detailed processing procedure is available elsewhere.

The dynamics of stable neck growth as summarized by Kuczynski is shown in the formula below [14]:

(1) $$\begin{array}{c} {\left(\frac{x}{a}\right)}^{n}=\frac{F\left(T\right)}{a^{m}}t, \end{array}$$

where *x* and *a* represent sintering neck and particle size, respectively, *t* is the sintering time, and different *n* represents different main diffusion mechanisms. The fourth state is not taken into account because of the presence of liquid aluminum. From Figure 6, it can be seen that there is a linear relationship between log(*x*) and log(*t*), and the slope is 0.1361; the value of inverse slope equals 7.3, indicating that surface diffusion was the dominant diffusion mechanism, which was caused by conductance characteristic of metals. Al and Ti are electrical conductors; the eddy current loss induced by the external microwave fields constitutes the main loss mechanism. This eddy current is along the particle surface layer with the thickness about 1 *μ*m and will probably provide an additional driving force for mass diffusion on the particle surface. Therefore, on the Al and Ti particle surface, the mass transformation process was promoted preferentially.

**Figure 5:**
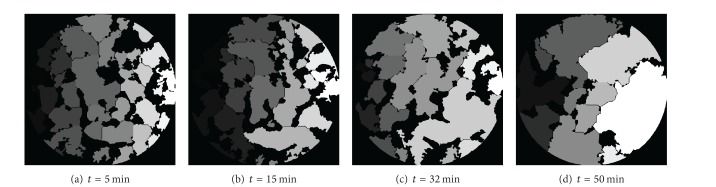
Microstructure of the same cross section using the watershed algorithm.

**Figure 6:**
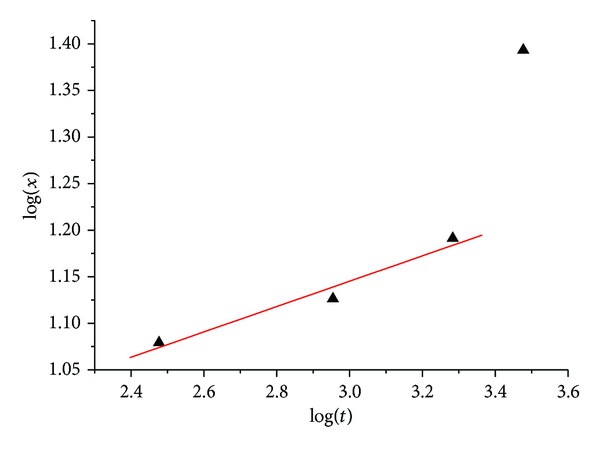
Double logarithm curve of mean neck size versus time.

**Figure 7:**
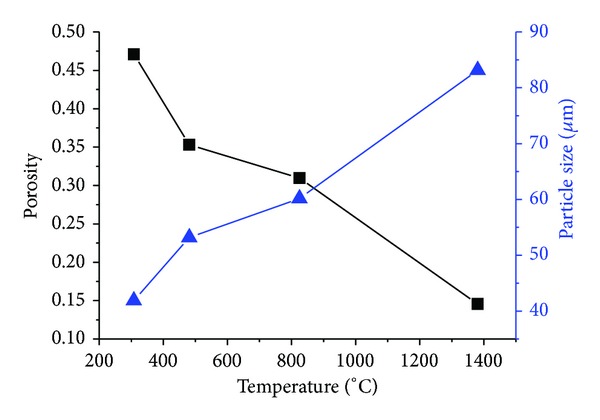
Mean particle size and porosity at different temperature.

Pores of a material reduce its mechanical properties, such as compressive strength, modulus, shear strength, and flexural strength [15]. With the movement of grain boundaries which is caused by the chemical potential difference at high-negative-curvature neck surfaces and grain boundaries, pores become fewer and neighboring particles become close to each other, with the disappearance of small particles, the number of particles is reduced, and the size is gradually increased (Figures 5 and 7). The temperature was measured by thermal infrared imager; the model was Thermo Tracer TH5104 (temperature range −10°C~1500°C, accuracy ±1.0%). As shown in Figure 7, the temperature increases to 1381.1°C, which is higher than the melting point of Al. It can be inferred that liquid aluminum was wetting on the surface of titanium and produced a capillary force. Densification process was accelerated by the force; it can be found in the rapid growth of particles in Figures 4(d), 5(d), and 7.

## 4. Conclusion

Based on the SR-CT technique, microstructure evolution of Ti-Al metal powder mixture was observed, and through the design of equipment and the introduction of excellent reconstruction method, the spatial resolution was firstly increased to 0.37 *μ*m/pixel during microwave sintering, which demonstrated that high-resolution experiments under electromagnetic and high temperature compound fields were feasible. The reconstructed 3D images provided good understanding of various sintering phenomena. Parameters that typically influenced the mechanical properties such as neck size, porosity, and average particle size were analyzed. The neck size-time curve was obtained and the neck growth exponent was 7.3, which indicated that surface diffusion was the main diffusion mechanism, and the reason was the eddy current loss induced by the external microwave fields providing an additional driving force for mass diffusion on the particle surface. From the reconstructed images and the curve of average particle size versus temperature, it was believed that the presence of liquid phase aluminum accelerated the particle growth and densification. In this exploratory study, only a small part of the data acquired by SR-CT was used for quantitative statistical analysis. The 3D reconstructed images represent the complete evolution of microstructure in high temperature and microwave compound fields, such as the smoothness and relative positions of particles, the volume shrinkage of the powder system, and collapse caused by thermal stress; all of these parameters are vital and need to be studied, and they contain the kinetic and thermodynamic information which directly determine the performance of the materials. The challenge is to decouple the information and find the key influence parameters used to calculation model, which makes the study on the sintering more thorough.

## Figures and Tables

**Table 1:** Relationship between size of view field and resolution.

Number	Size of view field/mm	Resolution/*μ*m
1	0.38	0.19
2	0.74	0.37
3	1.40	0.74
4	3.7	1.85
5	7.4	3.7
6	11	5.9
